# Drug trials in children: Doubts and dilemmas

**DOI:** 10.4103/0974-2069.43871

**Published:** 2008

**Authors:** Bharat Dalvi

A couple of months ago, a news item reported the tragic death of babies who were subjects of a clinical trial.[1] The report sensationalized certain elements viz. how the babies were from poor families, how a drug not to be used in children less than 18 years was administered, how informed consent was a sham and that children in developing countries were used as guinea pigs by the multinational pharmaceuticals. The scientific details about the drug under trial, the inclusion criteria for the study under consideration, the status of these babies before they were randomized, how many of the deaths occurred in the drug arm vs. the placebo arm and was the drug responsible or was it the disease which caused the death, all these were conspicuously absent. Possibly, these details would have taken away the “news” element from the report. There is no denying the fact that clinical trials in children need stringent scrutiny of design and very close monitoring during execution to avoid life threatening complications which formed the basis of this news item. Our hearts go out to the families who lost their beloved ones. However, the question that continues to bother us is whether or not clinical trials should be conducted in children?

Until a few years ago, almost all drugs used in children with heart disease, were justified/rationalized on the basis of data extrapolated from trials conducted in adults. This was despite the awareness that children are not miniaturized adults and newborns, a different subset altogether. With all the talk about evidence based medicine, in actual practice very little was done to obtain data on drug efficacy and safety in children. Studies have demonstrated that unlicensed and off-label drug use is frequent in hospitalized children, with up to 90% of newborns in intensive care units receiving either unlicensed or off label treatment.[2,3]

This raises some very basic questions related to drug therapy in children with heart disease. While prescribing in children and more so in newborns and preterms, do we really practice evidence based medicine? How many drugs that we prescribe in children for common cardiac ailments like congestive cardiac failure, hypertension, cyanotic spells have been subjected to the scrutiny of prospective, randomized, double blind trials which are considered as gold standard for proving their efficacy and safety. How many drugs have been subjected to studies to determine their optimal dosing and frequency of administration? Metabolic systems in newborns and infants are certainly very different, more so during the stress of cardiovascular decompensation often complicated by infection, anemia and renal and hepatic dysfunction. What then is the choice?

We have only two options. The first is to maintain status quo which though simple is not optimal since the current practices do not completely meet the standards of medical therapeutics. The other, more difficult option is to try and set up prospective, randomized, controlled clinical trials in children following very stringent criteria so that their safety is not compromised. Additionally, we need to study their pharmacokinetics in newborns and infants to optimize the dosing schedule. Pharmaceutical companies are reluctant to conduct such trials/studies in children because the pediatric market share is much smaller as compared to adult counterparts. Therefore investing in pediatric drug trials is financially less attractive. Moreover, there are difficulties in patient recruitment and blood sampling in addition to the ethical issues of subjecting children to new drugs.[4] However, none of these challenges seem to be unsurpassable. Conducting multicentric studies in specialty hospitals, studying lesser number of patients (as compared to what is expected in adult studies), giving incentives to pharmaceuticals for conducting such trials and partial state funding for such studies would be some of the options to overcome these difficulties.[4]

Lastly, do we need studies in children for every new drug that is introduced in the market? Certainly not. European guidelines on clinical investigations in children have made some recommendations in this regard.[5] We certainly need to evaluate drugs which are used for treating diseases which affect the children exclusively e.g. Kawasaki disease. Also, drugs for diseases which mainly affect the children or have a different natural history as compared to the adults, e.g. myocarditis, need to be assessed in children more aggressively. Lastly, the newer drugs which are being tested for diseases that occur in adults as well as children but for which there is no optimum treatment available e.g. the ones used in cardiac failure also need to be studied in children at an early stage of drug development.

The tragic episode that I referred to earlier once again brings into focus this debate of drug testing in children.
